# BanglaTaka: A dataset for classification of Bangladeshi banknotes

**DOI:** 10.1016/j.dib.2025.111853

**Published:** 2025-07-06

**Authors:** Md. Naimul Islam Nuhash, Sadia Akter

**Affiliations:** Department of Computer Science and Engineering, Daffodil International University, Daffodil Smart City, Birulia, Savar, Dhaka 1216, Bangladesh

**Keywords:** Bangladeshi banknotes, Machine learning, Deep learning, Computer vision, Image analysis, Taka classification

## Abstract

Automated classification of banknotes is essential for financial security and e-transaction systems. Conventional manual authentication is time-consuming and error-prone, which necessitates automated systems. This article introduces BanglaNotes, a benchmark dataset for Bangladeshi banknote denomination classification. The dataset contains 5073 images of Bangladeshi paper currency of nine denominations (2, 5, 10, 20, 50, 100, 200, 500, and 1000 BDT), with high quality and consistent representation. Every image in the dataset is labeled according to its denomination. The dataset is suitable for training and testing machine learning algorithms for currency classification and it supports research in financial automation and deep learning-based classification algorithms. It also offers a benchmark for developing robust algorithms for banknotes' real-life applications in banks, mobile payment systems, and ATMs. The dataset is publicly released to promote innovation and further research in banknote classification and recognition.

Specifications Table

**Table:** 

Subject	Financial Technology, Image Analysis
Specific subject area	Computer Vision, Deep Learning, Machine Learning, Image Classification, Image Processing, Financial Automation
Type of data	Images (JPG)
Data collection	The process of data collection took place from September to November 2024 while ensuring environmental variation and real-world applicability. The BanglaTaka Dataset contains 5073 high-resolution images of Bangladeshi banknotes in nine denominations, gathered from varied sources including small shops, banks, markets, homes, students' pocket money, and street vendors. Images were captured under different real-world conditions including lighting, viewing angles, and times of day variations. The images were taken by smartphone cameras, including iPhone 12 Pro, Samsung M31, Realme C25s, Samsung M21, and Xiaomi Redmi Note 13.
Data source location	Images in this dataset were collected from different areas of Bangladesh, including: Dhaka: 23.8103° N, 90.4125° E Alamdanga: 23.7627° N, 88.9438° E Rajshahi: 24.3746° N, 88.6004° E Khulna: 22.8373° N, 89.5400° E Kushtia: 23.9089° N, 89.1222° E Chattogram: 22.3752° N, 91.8349° E Sylhet: 24.9048° N, 91.8600° E Feni: 23.0128° N, 91.3965° E
Data accessibility	Repository name: Mendeley Data [16] Data identification number: 10.17632/3cv2sypkkh.1 Direct URL to data: https://data.mendeley.com/datasets/3cv2sypkkh/1

## 1 Value of the Data

• Comprehensive Banknote Dataset: BanglaTaka contains 5073 high-resolution images of Bangladeshi banknotes across nine denominations, providing a valuable asset for machine learning-based currency classification. The dataset provides a reliable benchmark for extensive training and testing of currency classification models, thereby fostering innovation in the sector.
• Facilitating Financial Automation: The data is well-suited for training deep learning models, improving accuracy in automated banknote recognition in banking, ATMs, and mobile payment systems.
• Strong and Diverse Representation: Recorded using several smartphones under diverse conditions, the data ensures preparedness for real-world settings. Preprocessing maximizes visibility by removing background noise and improving contrast.
• Extensive Uses: Facilitates financial stability studies, detection of forgery, and currency classification based on AI, employed in banking automation and online transactions.

## 2 Background

Paper currency is highly valuable in banking transactions, commerce, and financial stability, particularly in Bangladesh, where most of the people still use it as the principal medium of exchange. In the era of automatic technologies, monetary transaction automation becomes inevitable to ensure maximum efficiency in mass institutions, ATMs, banks, governmental agencies, and small businesses. Small institutions and individuals, especially the blind, also require inexpensive but efficient banknote classification and recognition solutions. Manual verification processes tend to be time-consuming, prone to human error, and wasteful, prompting the necessity of automation processes that ensure safe and seamless transactions.

Advances in artificial intelligence, particularly deep learning techniques such as Convolutional Neural Networks (CNNs), have revolutionized image-based classification issues. Various methods and algorithms have been proposed in banknote research to address fundamental issues like detection of counterfeiting [1,2], classification of new and old banknotes [3,4], identification of banknote denominations to assist the blind [5,6], country-specific classification of banknotes [7,8], and banknote defect detection [9,10]. The performance of these AI models, however, relies heavily on the availability of good quality and diversified datasets. To fulfill this need, the BanglaTaka dataset was developed, comprising 5,073 accurately labeled images of nine Bangladeshi banknotes.

Over time, currency notes may pick up noise as they degrade, presenting the challenge of being detected by systems as old, degraded, or worn-out banknotes [11]. To mitigate this constraint, we introduced diversity in our dataset by incorporating worn-out and new notes. The dataset was recorded using various smartphone cameras in real-world environments, keeping in view the variation of lighting, orientation, and environmental conditions for generalizability and robustness.

Existing publicly available Bangladeshi banknote datasets have several limitations in terms of real-world applicability and variability. The dataset considered by R Tasnim et al. [17] and described in [18] contains largely clean and idealized images with very limited variability as occurs in real-world conditions. Further, the dataset contains only eight denominations, reducing the overall completeness. Similarly, the dataset considered in [19] includes only 500 and 1000 Taka notes, excluding a large part of the currency. In another study, MJA Rafi et al. [20] introduced a dataset [21] in which most images have partial views of banknotes and are typically captured with cluttered backgrounds. Hence, the dataset is less suitable for counterfeit detection, which requires clean and complete visual information. Additionally, it lacks images of worn-out, torn, or defaced notes—features common in real-world financial transactions. Such limitations present significant challenges to the training of robust and generalizable machine learning models for works such as banknote recognition and counterfeit detection [25,26,28].

To address the limitations identified in existing publicly available datasets, we have developed and presented a large-scale dataset of Bangladeshi banknotes that emulates a wide range of real-world conditions. This database includes clean and dirty banknotes, recording the variability typically present in daily use, for example, circulation wear, folding, staining, and partial fading. All the images are background-free and display the full visual features of every note, which is crucial for a variety of research tasks—for example, ensuring full visibility of security features, denomination indicators, and sophisticated design patterns that are essential for accurate classification, recognition, and counterfeit detection. Unlike current datasets, our collection includes all denominations that are currently in circulation, making it easier to develop more stable and generalizable machine learning models. Furthermore, the dataset provides sufficient resources not only for denomination recognition but also for a broad range of currency research tasks, including serial number recognition, counterfeit detection (when paired with counterfeit note datasets), and automated teller machine (ATM) currency acceptance systems.

This dataset is a valuable resource for AI-driven currency identification and finance automation systems to assist in alleviating the need for manual verification and maximize banking, mobile money, and automated teller machine business efficiency. It is also a source of interdiscipline research in financial technology through the integration of computer vision and cutting-edge AI techniques for currency detection, counterfeiting, and digital financial security. With its offering of a standardized dataset, BanglaTaka offers a foundation for stable and scalable models of banknote classification to emerge, advancing the agenda on financial automation and digital payments.

## 3 Data Description

Automation of banknote classification can conveniently enhance the efficiency of sorting currencies, reduce the workload and save time. The BanglaTaka dataset includes all the paper currency notes of Bangladesh except for the 1 Taka note. The BanglaTaka dataset is a dataset for real-world usage.

Images were collected from diversified sources, including small shops, banks, markets, residential homes, street vendors, and pocket money of students, to provide diversified acquisition sources reflecting real currency usage. The dataset is consistently labeled into nine distinct classes corresponding to the nine denominations of banknotes (2, 5, 10, 20, 50, 100, 200, 500, and 1000 Taka). Images are organized by denomination, and files are renamed sequentially to facilitate easy identification and processing.

All the images are provided in JPG format to enable compatibility and the dataset is provided in the form of a ZIP file so it can be downloaded easily and incorporated smoothly in various machine learning and financial software applications. The breakup of the classes of the dataset is explained elaborately in Table 1 below.

**Table 1:** Dataset description with sample image.

## 4 Experimental Design, Materials and Methods

### 4.1 Experimental design

The BanglaTaka Dataset for the classification of banknotes was designed carefully to bridge the gap of a large dataset of Bangladeshi currency images from different real-world sources. The primary motive of this dataset is to facilitate machine learning models, particularly deep learning models like CNNs, to classify banknotes based on their denominations successfully.

**Fig. 1:**
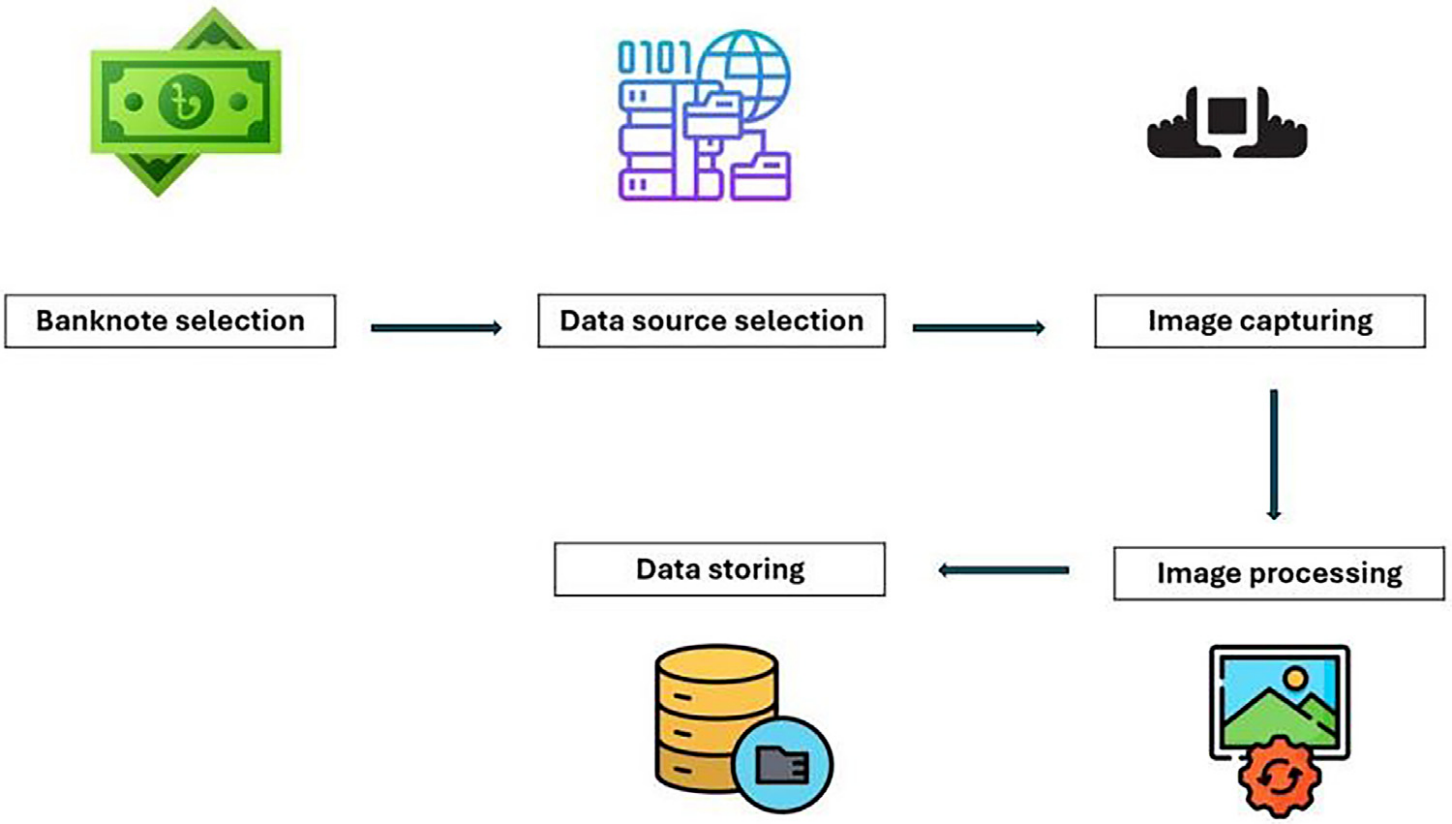
Key steps of gathering the dataset.

The dataset creation process is explained in the following section. The dataset was developed by adhering to some significant steps, as evident in Fig. 1, in order to ensure diversity, strength, and real-world applicability.

The first step was the selection of banknotes to be included in the dataset, ensuring all denominations except the 1 Taka note were covered. The source images were subsequently properly selected so as to include the maximum number of diverse images as well as their representation considering the locations of different banknote circulation status. Identified sources, images were later obtained using diverse smartphone cameras considering diverse lighting, angle conditions. Preprocessing entailed cropping, resizing, and contrast modification on images without affecting their originality. The images were subsequently stored systematically, categorized into nine classes according to denomination, and arranged in an organized fashion for easy retrieval and utilization.

### 4.2 Banknote selection

here are ten banknotes available in Bangladesh, from 1, 2, 5, 10, 20, 50, 100, 200, 500, and 1000 Taka. Among these, the most common ones used in day-to-day transactions are 5, 10, 20, 50, 100, 200, 500, and 1000 Taka notes. But the 1 and 2 Taka notes are rarely found.[12] In particular, the 1 Taka note has been significantly replaced by the 1 Taka coin and is not in common use. Since it was not available and was rarely in use, the 1 Taka note was excluded from the dataset. The dataset instead aims at the remainder of the banknotes, ensuring to capture the most frequently used denominations in all over Bangladesh extensively.

### 4.3 Data source selection

All the Bangladeshi notes in circulation do not consist of only new, fresh, and unused banknotes. Instead, they are made up of fresh, old, clean, and noisy banknotes. Repeatedly used notes are usually in wear and tear, which is in the form of soiling, stain of ink, crease, fading, and tear. These will be caused by extended handling, improper storage, and subjecting to different ambient conditions.

We collected banknotes images during our time spent in different regions of Bangladesh and observed regional variations in currency circulation, especially between the rural and urban regions. In big cities like Dhaka, Rajshahi, Chattogram, Sylhet and Khulna, higher denomination currency especially 500 and 1000 taka notes are commonly found, and while 2 and 5 taka notes are relatively scarce—shopkeepers often give candies instead of small change. On the other hand, rural areas like Alamdanga, village markets, and surrounding communities still use lower denominations widely, and most of our 2 and 5 Taka notes were acquired from these locations. Urban stores typically reject noisy or damaged notes, so citizens refrain from having them. However, rural shops and individuals such as farmers and laborers frequently use worn, torn, or stained notes, resulting in a wider variety of note conditions. In cities, fresh and clean banknotes are readily available since there is greater access to banking facilities. New banknotes are even retailed at a premium at Eid festivals in Dhaka [22]. Occupational habits also influence condition of notes—city office workers keep clean notes in wallets, while countryside workers like farmers or fishers keep notes in pockets or cover them in cloth, causing more physical damage [27]. These are regional and occupational differences that contributed importantly to the diversity of banknotes in our sample.

**Fig. 2:**
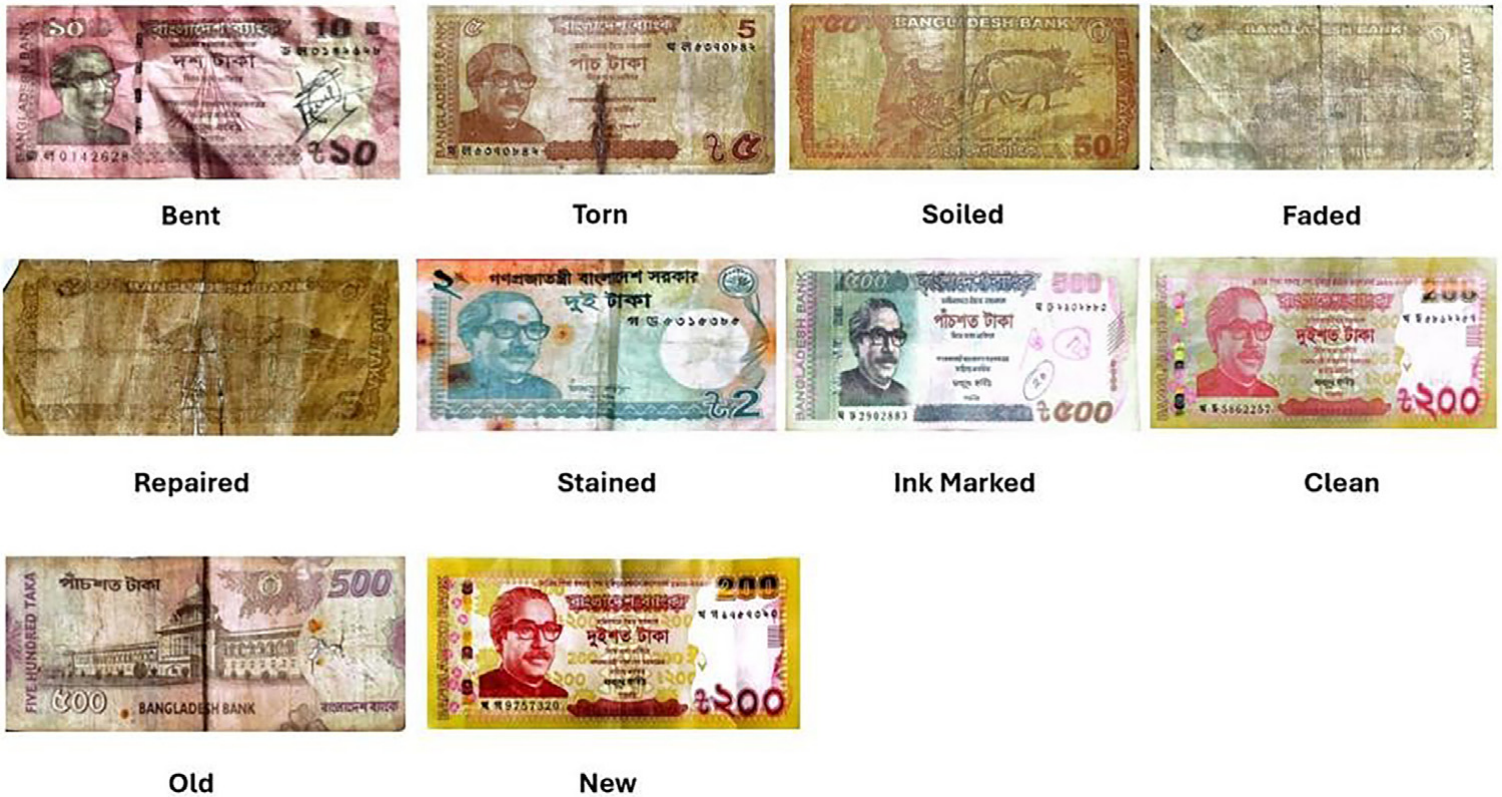
Sample of banknotes with different physical states.

To ensure a representative and representative dataset, we chose data sources carefully based on where banknotes are likely to circulate and experience varying levels of wear. Transaction environments, handling frequency, and exposure to the external environment were some of the key considerations. Relatively clean banknotes were sought from banks, moderately worn banknotes from small markets and shops, and highly worn banknotes from street vendors, households, and students' pocket money. Notably, 2 Taka and 5 Taka notes were mostly in bad physical condition. Fig. 2 presents banknotes in different physical conditions.

This strategic decision ensured a well-balanced dataset that fortified the model to detect real-world variations. By mixing new and old banknotes in the same data set, the data enhances the overallizability of machine learning models that makes them more accurate in real-world financial transactions.

### 4.4 Image capturing

The efficient currency recognition system must have the ability to identify banknotes from both sides and under various conditions, i.e., new, old, and noisy notes [13]. To that end, images were captured using the rear cameras of different smartphones of varying camera specifications and configurations:

• iPhone 12 Pro: 12 MP (wide, f/1.6) + 12 MP (ultrawide, f/2.4) + 12 MP (telephoto, f/2.0, 2x optical zoom)
• Samsung M31: 64 MP (wide, f/1.8) + 8 MP (ultrawide, f/2.2) + 5 MP (macro, f/2.4) + 5 MP (depth, f/2.2)
• Realme C25s: 48 MP (wide, f/1.8) + 2 MP (macro, f/2.4) + 2 MP (depth, f/2.4)
• Samsung M21: 48 MP (wide, f/2.0) + 8 MP (ultrawide, f/2.2) + 5 MP (depth, f/2.2)
• Xiaomi Redmi Note 13: 50 MP (wide, f/1.8) + 8 MP (ultrawide, f/2.2)

Images were collected randomly using multiple smartphones, without maintaining any fixed ratio or predefined quota for the number of images captured per denomination by each device. However, to ensure adequate diversity and representation, each device contributed at least 200 images to the dataset. Among these, the Samsung M31 was used to capture the largest portion of the dataset.

Some of the photos were taken with the flash on and some were ambient light-based. Some photos in the dataset were taken under different lighting conditions, such as natural daylight, artificial light (e.g., tube lights), and low-light conditions, i.e., nighttime conditions. Some images were taken using the default camera applications of the smartphones, and some were taken directly and scanned through CamScanner.

Both sides of every banknote were photographed separately to enhance recognition potential. Images were taken in two orientations: the normal readable orientation (upright) and upside down reversed, offering full data coverage for improved model performance.

## 5 Image processing

The performance of a recognition system can be significantly enhanced by applying preprocessing techniques. For example, recognizing worn banknotes in a note identification system requires essential preprocessing steps [14]. To maintain consistency across images captured with different smartphones under varying environmental conditions, the preprocessing pipeline in a standard form was adopted to carry out normalization. The background noise, which included hand, table, or irrelevant objects, was removed through manual and semi-automatic cropping to ensure the banknote was clearly highlighted in each image. This step enhanced the framing consistency and improved the visual clarity of relevant features. In addition, to resolve orientation inconsistencies resulting from both vertical and horizontal captures, all the images were reoriented to a standard horizontal format. The images were not resized to a fixed resolution so that their original sizes are preserved for any application cases demanding size-dependent analyses. The processed images were systematically sorted into denomination-specific folders to support organized dataset access and facilitate analysis. To improve the dataset's usability and ensure uniformity for training, we applied several preprocessing techniques and tools.

• Software Used: We used CamScanner to enhance the image quality. For cropping unnecessary parts of the images, we used laptop-based editing software, the default mobile editor, or CamScanner, depending on the need.
• Image Selection: A rigorous process of image selection and quality checking was carried out to preserve the integrity and usability of the dataset. As images were captured by multiple smartphones under diverse environmental and lighting conditions, numerous inconsistencies and artifacts were anticipated. During the image capture phase, a few challenges arose—e.g., such as currency notes being partially obscured by surrounding objects, blurred due to camera instability during manual capture, or disrupted by environmental factors like wind or background interference. Images that were very much blurred, not well aligned, or with blocked or incomplete views of the banknote were eliminated. Only clear and complete images that well preserved the visual characteristics of the bank notes were chosen to undergo further preprocessing and be included in the dataset. To ensure high-quality downstream tasks, we used the following selection criteria:

• Excessive noise: Images exhibiting significant interference—such as mobile flashlight glare, harsh shadows, or strong reflections—were discarded, as such artifacts can obscure key visual features of the banknotes.
• Blurred or low-resolution images: Any image that appeared excessively blurred, pixelated, or out of focus to the extent that the denomination or security features were not clearly visible was excluded.
• Incomplete notes: Images that did not show the full banknote—such as those with cropped edges or occluded parts—were rejected. This included photos where parts of the note were blocked by hands, writing, or other objects.
• Duplicate or near-duplicate images: Redundant images showing the same note from nearly identical angles and conditions were also filtered out to maximize diversity within the dataset.

**Fig. 3:**
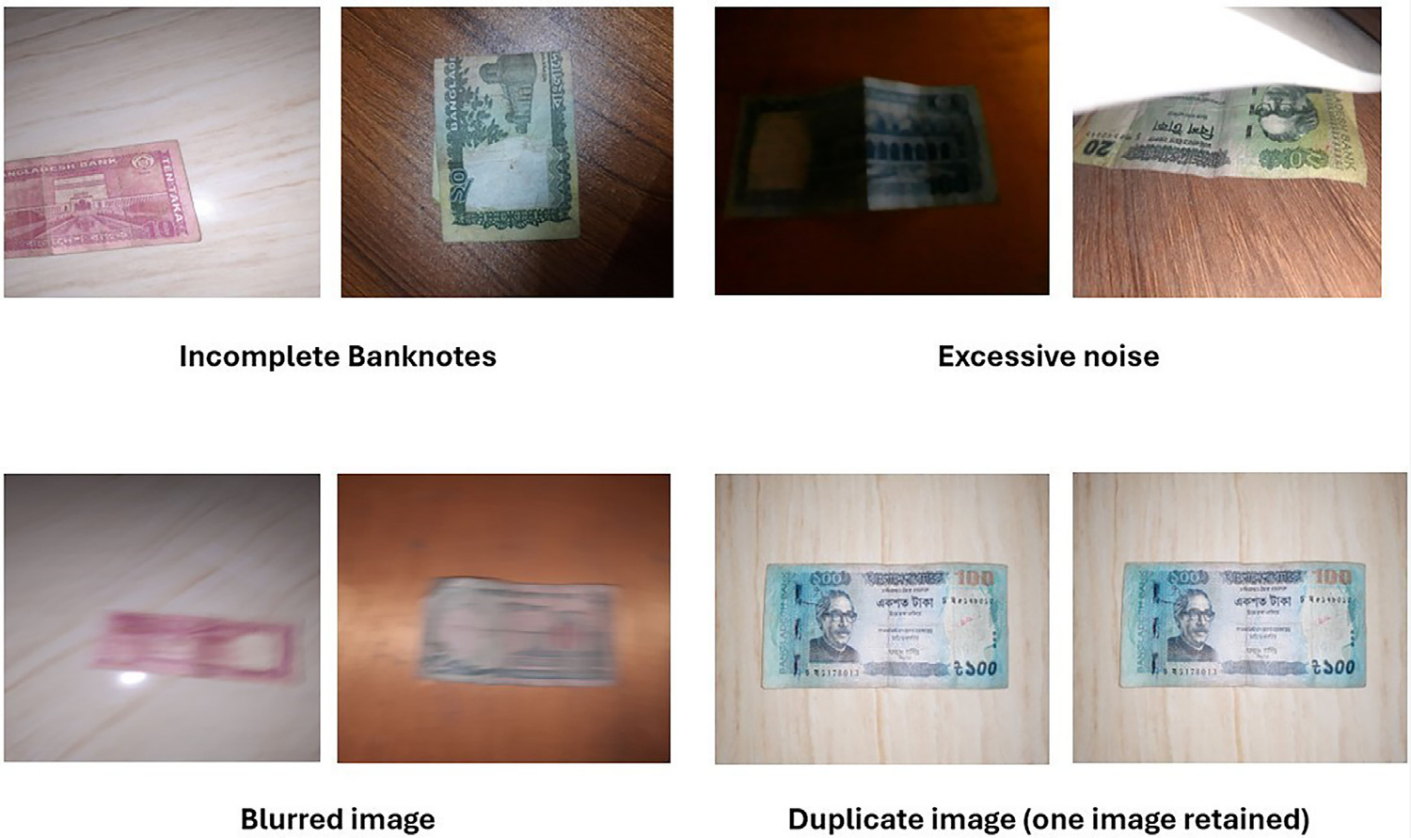
Excluded images due to quality issues.

Only those images that passed this quality control stage—i.e., those displaying complete, clearly visible, and interpretable banknotes—were selected for further enhancement and cropping. This was necessary to ensure that the final dataset is visually representative and actual currency conditions so that it can facilitate correct and sound machine learning applications. Fig. 3 presents some examples of rejected images that were excluded during the quality control process due to issues such as blurriness, obstruction, or poor framing.

• Image Enhancement: Image enhancement is the process of improving the visual quality of an image to make it more suitable for specific applications [23], i.e., machine learning and pattern recognition. Enhancement was necessary to highlight the key features of each banknote. We employed a combination of enhancement techniques, including contrast stretching, color correction, and brightness adjustment.

• Contrast stretching was used to expand the range of intensity values in images, making the subtle patterns and text on the banknotes more visible.
• Color correction helped balance color tones and remove any distortions caused by lighting inconsistencies or mobile camera sensors.
• Brightness adjustment was applied to ensure that underexposed or overexposed regions were corrected, improving the legibility of security features and printed content.

**Fig. 4:**
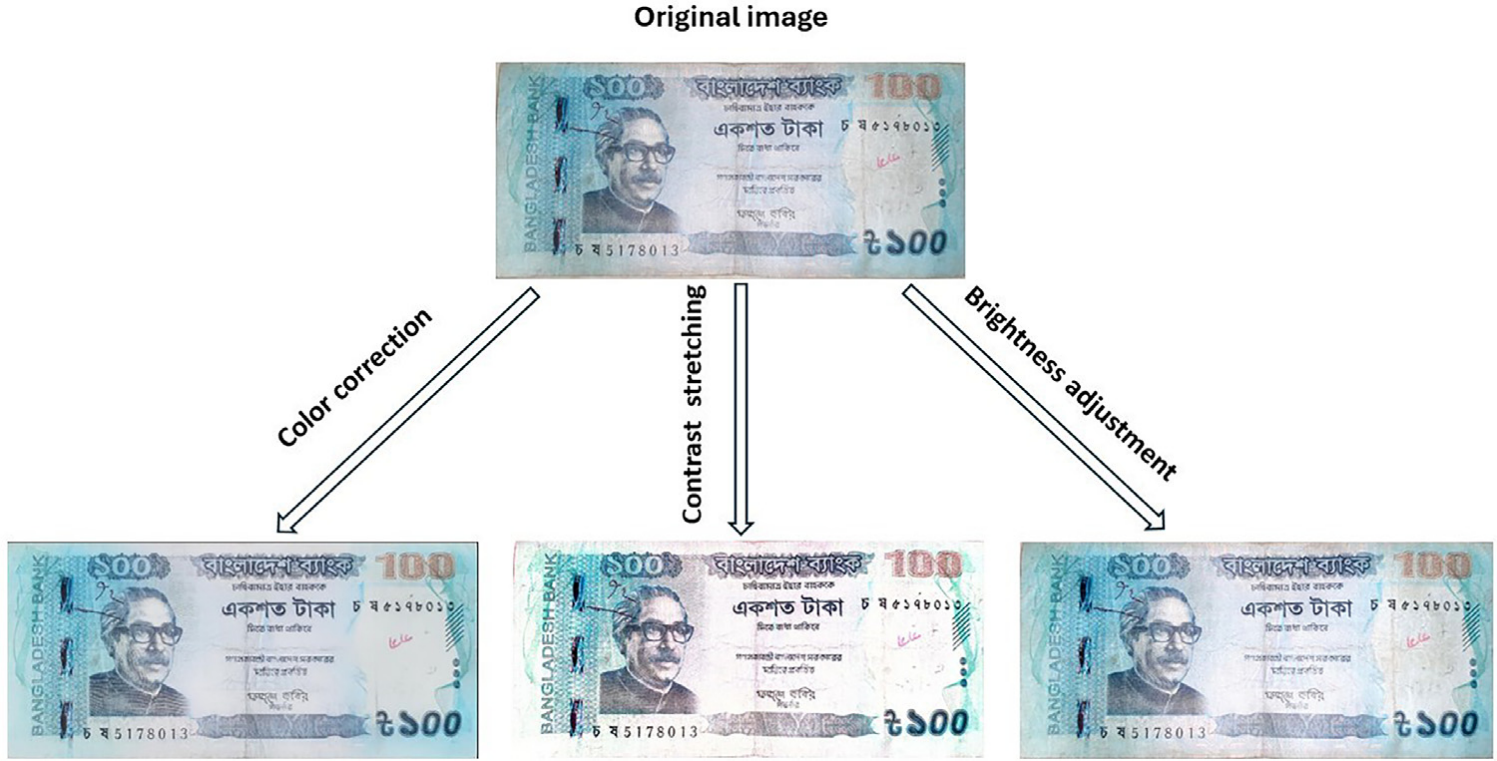
Application of image enhancement

All these processes together improved image interpretability, especially in cases where original images were of poor contrast, low light, or color imbalance. CamScanner, regular mobile editors, and computer-based photo editing software were the tools employed to gain these improvements. Fig. 4 illustrates the use of image enhancement techniques on a Bangladeshi 100 Taka note, which includes color correction, contrast stretching, and brightness adjustment.

• Cropping: Image noise is largely an undesired byproduct of image acquisition [24]. Cropping is a crucial preprocessing step to delineate the region of interest—in this instance, the banknote—from undesirable background objects. During data collection, images were captured in various environments, and as a result, raw images had background clutter such as tables, hands, or other objects.

**Fig. 5:**
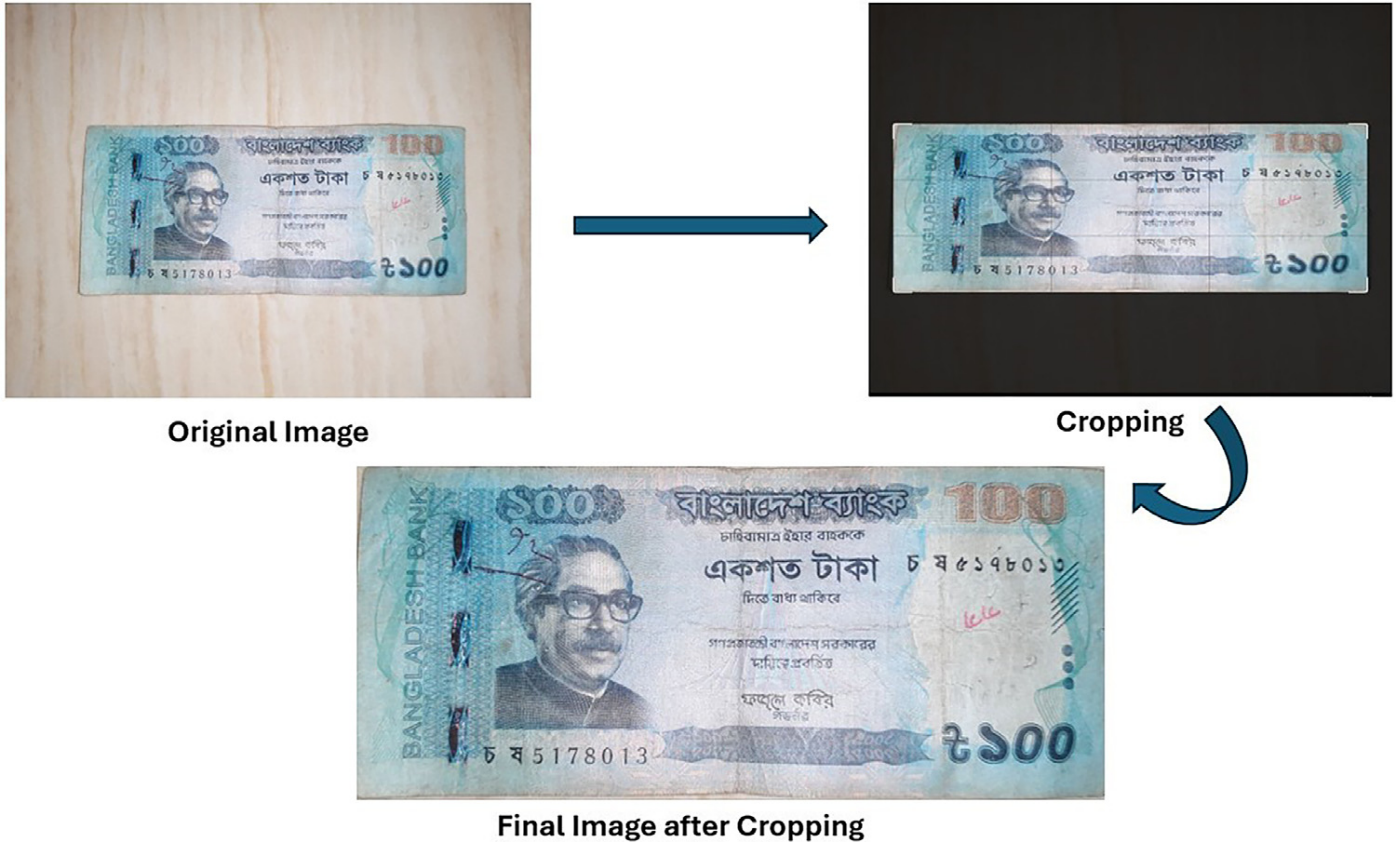
Cropping of raw image to extract clean banknote region.

To correct this, we used manual and semi-automated cropping using CamScanner, mobile photo editing software, and laptop-based applications. The aim was to remove unwanted parts of the image while leaving the entire visible section of the banknote intact, with important visual features such as security threads, denomination labels and design patterns still intact. By eliminating distracting backgrounds, cropping improved the consistency of the dataset significantly and focused the learning task of machine learning models on the actual currency notes. An illustration of the cropping process is provided in Fig. 5.

### 5.1 Data storing

After capturing and preprocessing the images, the next task was to assemble them in a structured dataset. The images were placed in preexisting folders labeled according to their respective denominations. Each of the images was renamed in a naming format composed of the denomination and an image number (e.g., 1000 Taka 001). This nomenclature system made it simple to track every note for editing, inclusion, or exclusion in the future. All images were renamed in an instant with the ``Rename & Organize'' application, without manual renaming, saving time. Images in the dataset were saved in JPG format since most image processing and machine learning programs support it.

To facilitate easier sharing and uploading, the dataset was archived in a ZIP file. The ZIP file extension was chosen because it compresses the files efficiently without loss of quality for the images. It also consolidates a number of files into one archive, which makes data transferring and storing more convenient [15]. ZIP files are system-friendly, large-file-friendly, and also provide the option to encrypt, which makes them perfect for storing and sharing datasets like this.

## Limitations

The dataset consists mainly of scanned images taken in controlled environments. Therefore, the system may not be that effective for complex or dynamic backgrounds. Uneven representation of denominations in the dataset may affect the generalization capability of the model across all categories. The system focuses only on Bangladeshi currency, which restricts the applicability of the system in scenarios requiring multi-currency recognition or broader global use cases. The dataset is primarily designed for denomination classification. Although the notes in the dataset are authentic banknotes, it does not include counterfeit samples or security-specific imaging techniques (e.g., ultraviolet imaging, watermark visibility). Therefore, it is currently unsuitable for currency authentication tasks. Future expansions could enhance the dataset’s utility by incorporating forged banknote samples and images captured under specialized lighting conditions, enabling counterfeit detection and improving real-world applicability.

## Ethics Statement

The proposed dataset does not involve human experiments, nor any such experiments involving animals, nor do we involve social media data. We affirm that our research complies with the ethical requirements established by “Data in Brief”.

## CRediT Author Statement

**Md. Naimul Islam Nuhash:** Conceptualization, Methodology, Software, Formal analysis, Resources, Investigation, Data Curation, Writing - Original Draft. **Sadia Akter:** Conceptualization, Software, Resources, Investigation, Data Curation.

## Data Availability

Mendeley DataA Diverse Image Dataset for Bangladeshi Currency Recognition (Original data).
